# Predicting Polymers’ Glass Transition Temperature by a Chemical Language Processing Model

**DOI:** 10.3390/polym13111898

**Published:** 2021-06-07

**Authors:** Guang Chen, Lei Tao, Ying Li

**Affiliations:** 1 Department of Mechanical Engineering, University of Connecticut, Storrs, CT 06269, USA; guang.chen@uconn.edu (G.C.); lei.tao@uconn.edu (L.T.); 2 Polymer Program, Institute of Materials Science, University of Connecticut, Storrs, CT 06269, USA

**Keywords:** polymer informatics, machine learning, glass transition temperature, high-throughput screening, recurrent neural network

## Abstract

We propose a chemical language processing model to predict polymers’ glass transition temperature ($T_{g}$) through a polymer language (SMILES, Simplified Molecular Input Line Entry System) embedding and recurrent neural network. This model only receives the SMILES strings of a polymer’s repeat units as inputs and considers the SMILES strings as sequential data at the character level. Using this method, there is no need to calculate any additional molecular descriptors or fingerprints of polymers, and thereby, being very computationally efficient. More importantly, it avoids the difficulties to generate molecular descriptors for repeat units containing polymerization point ‘*’. Results show that the trained model demonstrates reasonable prediction performance on unseen polymer’s $T_{g}$. Besides, this model is further applied for high-throughput screening on an unlabeled polymer database to identify high-temperature polymers that are desired for applications in extreme environments. Our work demonstrates that the SMILES strings of polymer repeat units can be used as an effective feature representation to develop a chemical language processing model for predictions of polymer $T_{g}$. The framework of this model is general and can be used to construct structure–property relationships for other polymer properties.

## 1. Introduction

Glass transition temperature ($T_{g}$) of polymers is an important physical property, which has been studied extensively in polymer science and engineering [1,2,3,4,5,6]. $T_{g}$ characterizes a second-order phase transition over which polymers can change between a rubbery state and a glassy state with Young’s modulus ranging from MPa to GPa [7]. Thus, $T_{g}$ values determine the ease of processing during manufacturing and the application ranges in their deployment. Theoretical studies have provided many chemical and physical insights into $T_{g}$ of polymers, from thermodynamics to kinetics theories [4,8,9,10]. It is well known that $T_{g}$ value is dependent on the chain mobility or free volume of a polymer [9]. Specifically, it depends on the molecular weight, cross-links, side groups, and chain ends of a polymer. Though theoretical studies have offered critical understandings of polymer’s glass transition, it is still deficient for accurate predictions of $T_{g}$ of general polymers and not effective for inverse polymer design.

While experiments and computer simulations, e.g., molecular dynamics [11,12,13,14], are feasible approaches to quantify the $T_{g}$ of polymers, the data sizes, and sample types that can be handled by these methods are usually limited due to the significant cost in experimental or computational measurements. Nonetheless, these measurements have provided a diversified polymer database that can be leveraged by data-driven studies.

In general, data-driven studies try to construct a mapping relation between the polymer’s chemical structures to the corresponding $T_{g}$ or other properties [15,16,17,18]. The development of quantitative structure-property relationships (QSPR) of polymers have significantly benefited quantitative predictions of polymer’s $T_{g}$ [19,20,21]. This type of studies has also been called polymer informatics [22,23,24,25]. Recently, thanks to the advances in computing power and the availability of big data, machine learning (ML), especially deep learning (DL), has attracted enormous attentions in various scientific fields and indeed brought in numerous breakthroughs in material science [17,26,27,28,29,30,31] and drug discovery [32,33,34,35]. However, it is not the case when it comes to polymer science and engineering, such as polymer’s $T_{g}$ prediction and other properties.

The main reason is that the database of polymers with high quality is very limited. In polymer literature, the database in most of previous studies were under a few hundreds or even less [36]. Therefore, DL models were not widely applied in these studies. It is because DL models usually have a large amount of parameters and thus are easy to over fit if trained on a limited amount of data [37]. Nevertheless, there are a few previous studies employing DL for polymer’s $T_{g}$ prediction. For example, the deep neural network (DNN) model [37,38] and convolutional neural network (CNN) model [39] have been recently employed to correlate polymer’s chemical structure (monomers) and its $T_{g}$, although the data size in these studies are rather limited. Very recently, Nazarova et.al. studied the dielectric property of polymers using the recurrent neural network (RNN) on 1200 polymers, though the model was only tested on 60 samples [40]. Note that DL models have widely been used for another type of tasks without labeled polymer properties, i.e., molecular generation using deep generative models [29,31,41,42,43,44]. This kind of tasks is to use deep generative models to learn the conditional probabilities of the SMILES strings [45,46,47] of organic molecules. The task in this study is a supervised learning of the syntax of SMILES strings for polymer’s $T_{g}$ prediction.

To develop DL models with good performances for polymer $T_{g}$ prediction, a large amount of polymer data is necessary since DL models usually have a large number of parameters and thus are easy to overfit. Recently, a polymer database, called PolyInfo [48,49], has attracted much attention as it contains about 7000 homopolymers with experimentally measured $T_{g}$ values. However, since the database uses the SMILES strings of the polymer repeat units for polymer representation, the inclusion of polymerization point ‘[*]’ in the SMILES strings brings several difficulties for common cheminformatics packages to generate molecular descriptors or fingerprints, which have been extensively used in polymer informatics [25,30,50]. For cheminformatics packages like AlvaDesc [51], the SMILES strings with ‘[*]’ cannot be processed. While some other packages such as RDKit [52] can process this type of SMILES strings for descriptor generation, not all of them are available as the symbol ‘[*]’ is an unknown element for them to process, though RDKit can still generate molecular fingerprints for the SMILES with ‘[*]’. This is probably the reason why the monomers of polymers have been adopted for molecular descriptors/fingerprints generation as they are very easily processed, although it is criticized that monomers are not enough for polymer’s morphological representation [25,37,53,54].

**Figure 1:**
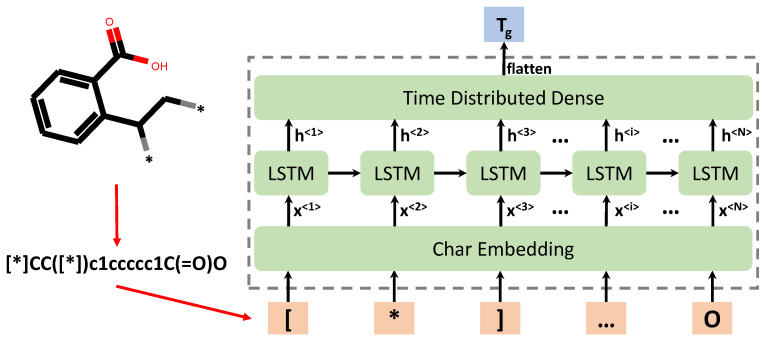
Schematic of the computational framework for chemical language processing model, in which a polymer’s repeat unit is first represented by its canonical SMILES string, which is further separated into individual chars as inputs of the RNN model. In the RNN model, light orange color denotes the input chars of SMILES string of polymer’s repeat units; green color denotes the intermediate layers, including embedding layer, LSTM layer and dense layer; the light blue color denotes the final output layer of the predicted $T_{g}$ values of polymers.

Here, in order to avoid this deficiency and use the polymer representation directly, we propose a chemical language processing model which is purely linguistic-based on the SMILES strings. The idea is to consider the polymer’s repeat unit (SMILES) as sequential data at the character level. It is then processed by a polymer embedding layer and the RNN for DL model development [55,56,57]. RNNs have enjoyed great success in, e.g., music processing, and language translation [58,59]. In the field of cheminformatics, they have also been widely applied as deep generative models for molecular generations [29,31,41,42,43]. A majority of RNN generative methods have been integrated in the generative adversarial network (GAN) and variational autoencoder (VAE) for molecule generation. For example, After Yu, Lantao, et al. [60] have used the RNN variant—LSTM in GAN to generate sequences, Guimaraes, et al. [61] utilized the same strategy to generate molecules with desirable properties. And then based on which Lengeling et al. [62] present their Objective-Reinforced Generative Adversarial Network for Inverse-design Chemistry (ORGANIC)—which is able to generate novel molecules such as with melting points above 800 K. If integrated in VAE, another RNN variant—GRU has also been utilized for molecule generation. Gø’mez-Bombarelli, et al. [63] have implemented a encoder RNN (GRU) to convert molecules into a latent space vector, and then convert it back to molecule smiles with a decoder RNN (GRU). Operations in latent space allow the decoder RNN to generate novel molecules with optimized properties. To improve the validity rate (valid decoded molecules to the total decoded molecules), Chaochao Yan, et al. [64] have built a VAE model with the bi-directional GRU and uni-directional GRU being the encoder and decoder. Their valid expression rate for the generated molecules is more than 90%. These RNN processing SMILES for molecule generations have been developed extensively, but few studies have been focused on RNN processing SMILES to predict molecule properties [33,41]. To our best knowledge, this work is the first to apply purely linguistic-based (SMILES) DL models for polymer’s $T_{g}$ prediction. The schematic of this model for $T_{g}$ prediction is given in Figure 1, which will be introduced in detail in the later sections. The results show that this method is a good alternative to the conventional methods based on molecular descriptors or fingerprints.

The remaining of the paper is organized as follows. The computational methodology of the chemical language processing model is presented in Section 2. Specifically, the database and feature representation of polymers, the char embedding, RNN, and DL models are described in detail. The ultimate architecture of the model and its performance tests are given in Section 3. Several aspects of the chemical language processing model are further discussed in Section 4. Finally, the paper is concluded by remarks in Section 5.

## 2. Computational Methods

### 2.1. Database and Feature Representation

**Figure 2:**
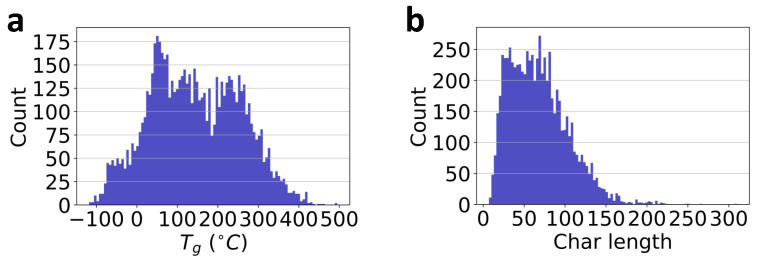
Database visualization. (**a**): the distribution of $T_{g}$ values in the database, PolyInfo; (**b**): the distribution of the length of chars in the SMILES strings of the polymer’s repeat units.

There are 7372 polymers in total in the current database. The respective $T_{g}$ count distribution is presented in Figure 2a. As mentioned previously, the SMILES strings of polymer repeat units are employed for polymer representation. Note, however, that the general SMILES string may not be unique for molecular representation. For example, ‘C1=CC=CC=C1’ and ‘c1ccccc1’ are all valid SMILES strings of benzene. To eliminate the inconsistency in the general SMILES representation, all the SMILES strings of polymer’s repeat units in the database have been processed to the corresponding canonical SMILES string using the RDKit cheminformatics package [52].

With this large database of polymers and SMILES string representation for polymer repeat units, the prediction of polymer’s $T_{g}$ is considered as a chemical language processing problem using the RNN. A significant advantage of this method is that no molecular descriptors or fingerprints are generated for ML model development to get around the restrictions on SMILES in descriptor generation.

In the natural language processing field, word-level or char level models can be applied as sentences are composed of words [65,66]. However, for polymer repeat units, only ‘word’ structure exists, i.e., SMILES strings. Thus, in this work, the char level RNN model is formulated to learn the chemical language of polymers in the SMILES notation. As shown in Figure 1, the pre-processing step is to split the SMILES string into a list of individual chars, which are then tokenized into integers and fed into the embedding layer of the DL model.

### 2.2. Char Embedding

Generally, in ML model development, the inputs are usually represented in digit numbers so that mathematical models can be constructed [67]. It is the same case for natural language processing. Two methods are usually used for word or char encoding in previous studies, namely one-hot encoding and categorical encoding. In this work, the latter is adopted for char encoding using the position it appears in the char lexicon. The whole list of chars contained in the polymer database is alphabetically as follows:

char lexicon = {‘#’, ‘%’, ‘(’, ‘)’, ‘*’, ‘+’, ‘−’, ‘0’, ‘1’, ‘2’, ‘3’, ‘4’, ‘5’, ‘6’, ‘7’, ‘8’, ‘9’, ‘=’, ‘B’, ‘C’, ‘F’, ‘G’, ‘H’, ‘I’, ‘K’, ‘L’, ‘N’, ‘O’, ‘P’, ‘S’, ‘T’, ‘Z’, ‘[’, ‘]’, ‘a’, ‘b’, ‘c’, ‘d’, ‘e’, ‘i’, ‘l’, ‘n’, ‘o’, ‘r’, ‘s’}

In the current database, the total number of characters in the list is 45. Consequently, any character in the list can be represented by an integer number in the range of 0 to 44 following the python index rule [68]. Therefore, any SMILES string can be represented by a vector composed of the index number of its individual chars. For example, the numeric representation of polyethylene ‘[*]CC[*]’ is [32,4,33,19,19,32,4,33]. In our polymer database, since the length of the SMILES strings are not the same or uniformly distributed as shown in Figure 2b, to accelerate the ML model development using batch training, a constant length has to be prescribed for the inputs. Another reason is to shorten the sequence length for the next LSTM layer to reduce training difficulties, as longer sequences may result in gradient vanishing or exploding problems during back-propagation. As a result, polymers with longer SMILES strings than the critical length will be truncated; while polymers with short strings will be padded with zeros in the trailing locations. In this database, over 82.1% polymers have shorter SMILES strings than 100; while about 91.2% polymers have shorter SMILES strings than 120. Thus, this number is considered as a hyperparameter in the ML model development to meet the trade-off between accuracy and computational efficiency.

Despite simple and clear, this encoding algorithm may not well represent similarities between words or chars. Therefore, this feature representation alone is not enough for meaningful feature extraction and for ML model development with good performance. In previous work [69], the authors tested DNN model performance just on integer-encoded vector by ASCII code for SMILES, the accuracy was very poor (accuracy score was about 0.53). It has been shown using word embedding can improve the model performances in natural language processing [70,71]. The objective of word/char embedding is to transform the one-hot or categorical encoding of words/chars into a new shorter yet dense vector with useful language meanings, which is learned by the DL model during model training. Hence, an embedding layer is adopted as the first layer of the chemical language processing model following the input layer, as shown in Figure 1. The purpose is that by applying an embedding layer, meaningful chemical information can be learned and passed to the recurrent neural network so that good performance can be achieved.

### 2.3. Recurrent Neural Network

**Figure 3:**
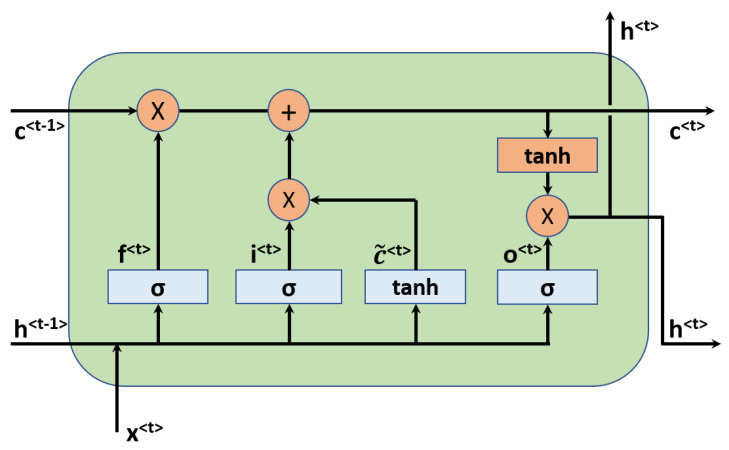
The LSTM unit used in the recurrent neural network.

The key idea of RNN is to use hidden variables to pass information from early states to later states for sequential data that has temporal dependencies [72]. RNNs have been the driving force in natural language processing, such as language translation and speech recognition. The simplest RNN unit is the so-called vanilla RNN, which suffers from gradient exploding or gradient vanishing problems in practice [72]. Therefore, more advanced units have been developed to build robust models, such as the Long Short-Term Memory (LSTM) unit [73] and the Gated Recurrent Unit (GRU) [74], both of which have been the golden standards of RNNs. The essential improvement is adding a cell state and gates to control the information flow in/out of the unit, in addition to the hidden state variables. In this work, the LSTM unit is employed in the RNN model. An illustrative figure for the LSTM unit is shown in Figure 3.

There are three gates in the LSTM unit, namely, the forget gate, input/update gate, and the output gate. Let the input be denoted by $x^{<t>}$ at the time step *t*, the hidden state and cell state variables be expressed by $h^{<t>}$ and $c^{<t>}$, respectively. The computational procedure in the LSTM unit is then:

(1) $$\begin{array}{c} \begin{array}{cc} f^{<t>} & =\sigma (W_{fh}h^{<t-1>}+W_{fx}x^{<t>}+b_{f})\\ i^{<t>} & =\sigma (W_{ih}h^{<t-1>}+W_{ix}x^{<t>}+b_{i})\\ o^{<t>} & =\sigma (W_{oh}h^{<t-1>}+W_{ox}x^{<t>}+b_{o})\\ {\tilde{c}}^{<t>} & =\tanh(W_{ch}h^{<t-1>}+W_{cx}x^{<t>}+b_{c})\\ c^{<t>} & =f^{<t>}\times c^{<t-1>}+i^{<t>}*{\tilde{c}}^{<t>}\\ h^{<t>} & =o^{<t>}\times \tanh\left(c^{<t>}\right) \end{array} \end{array}$$

where $f^{<t>}$, $i^{<t>}$, and $o^{<t>}$ are respectively the activated vectors for forget, update, and output gate. ${\tilde{c}}^{<t>}$ and $c^{<t>}$ are the input activated and the updated cell state, respectively. $W_{fh}$, $W_{fx}$, $W_{ih}$, $W_{ix}$,$W_{oh}$, $W_{ox}$, $W_{ch}$, $W_{cx}$, and $b_{f}$, $b_{i}$, $b_{o}$, $b_{c}$ are trainable weights and biases in the LSTM unit. The symbol ‘*’ denotes element-wise multiplication. σ is the nonlinear activation function such as sigmoid function, and tanh is the hyperbolic activation function.

Note that in addition to the unidirectional LSTM layer, the bidirectional LSTM layer has also been widely applied so that information can be passed from both early chars and later chars. Thus, the unidirectional and bidirectional LSTM networks are also considered for hyperparameter tuning.

### 2.4. DL Model Development

In this work, the DL model of chemical language processing is developed under the Tensorflow platform [75] mainly using the Keras package [76] to realize the aforementioned layers. To train and validate the chemical language processing model, the total database is split into a training dataset with 90% of the data and a test dataset with the remaining data because of a large database at hand. In the training process, the training dataset is further split into training and validation datasets by an 8:2 ratio to monitor model performance during training. The DL model is first trained on the training dataset and then evaluated on the unseen test dataset. Mathematically, the DL model seeks to find a prediction function $f:R^{d}\mapsto R$, which maps the inputs of chars in *d* dimensions to the $T_{g}$ value. The training process is equivalent to finding the optimal weights and biases by solving an optimization problem:

(2) $$\underset{w,b}{\arg\min}\;L\left(w,b\right)$$

where *w* and *b* are the weights and biases in the DL model, which keep updating by gradient descent scheme [77]. L(w,b) is the loss function, which is defined as:

(3) $$L\left(w,b\right)=\frac{1}{m}\sum_{i=1}^{m}{\left(y_{i}-{\hat{y}}_{i}\right)}^{2}$$

and the evaluation metric of the DL model on the test dataset is

(4) $$\mathrm{MAE}=\frac{1}{n}\sum_{i=1}^{n}\left|y_{i}-{\hat{y}}_{i}\right|$$

where *m* and *n* are the number of polymer samples in the training and test dataset, respectively. $y_{i}$ and ${\hat{y}}_{i}$ denote the real and predicted values of the $T_{g}$ of the *i*-th sample, respectively.

**Figure 4:**
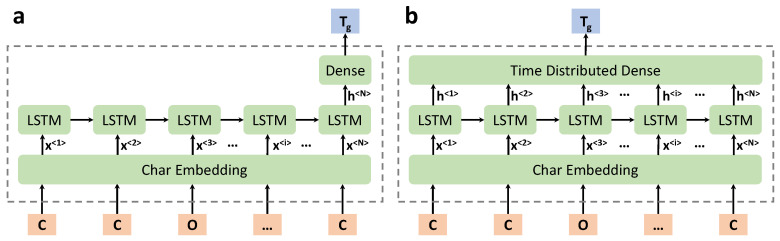
Two different types of DL model architectures. (**a**): Dense layer as an intermediate layer after the last LSTM unit of the previous LSTM layer; (**b**): Time Distributed Dense layer as an intermediate layer (flattened representation).

To develop an ML model with good performance, the grid search approach is usually adopted to tune the hyperparameters that lead to a relatively better model. The total hyperparameters considered in this work include:

(1) The maximum input length of the SMILES string (100 or 120);
(2) The length of the embedded vector (the output of the embedding layer);
(3) The type of LSTM layer (unidirectional or bidirectional);
(4) The number of LSTM layers;
(5) The number of hidden neurons for each LSTM units;
(6) The type of intermediate layers (dense layer or time distributed layer), as shown in Figure 4.

In the grid search of the optimal hyperparameters, the Adam optimization scheme [78] is adopted to minimize the loss function for weights and bias updates. In each case, the model is first trained on the training dataset, and then the prediction performance is evaluated on the test dataset using the mean absolute error (MAE) metric, which provides guidance on the selection of the optimal hyperparameters. The early stopping and checkpoints are employed to automatically cease training once comparable model performances are observed on the training and validation datasets.

## 3. Results

### 3.1. The architecture of the Chemical Language Processing Model

A series of chemical language processing models with various hyperparameters are developed according to the setup described in Section 2.4. Readers are referred to the Supporting Information for more details. It is observed that the DL model is relatively stable under different hyperparameters, with the MAE metric on the test dataset being in the range of 30∼34 °C. It is also observed that using the Time Distributed Dense layer (Figure 4b) may result in better model performance, which passes information out at each time step. While there is no obvious performance difference in DL models using unidirectional or bidirectional LSTM layers. The architecture of the optimal chemical language processing model is the one shown in Figure 4b.

Specifically, the char embedding layer receives an encoded char vector with a length of 120 and outputs an embedded vector of a length of 15 at each time step. In the next, two bidirectional LSTM layers are implemented with 60 hidden neurons for each layer. A Time Distributed Dense layer with 30 neurons follows the RNN (LSTM) layers subsequently. The final layer is a dense layer with only one neuron which denotes the predicted glass transition temperature $T_{g}$. All previous layers use the default activation functions while the final dense layer uses the linear activation function. Unless otherwise stated explicitly, the other parameters are following the default settings in the Keras package.

**Figure 5:**
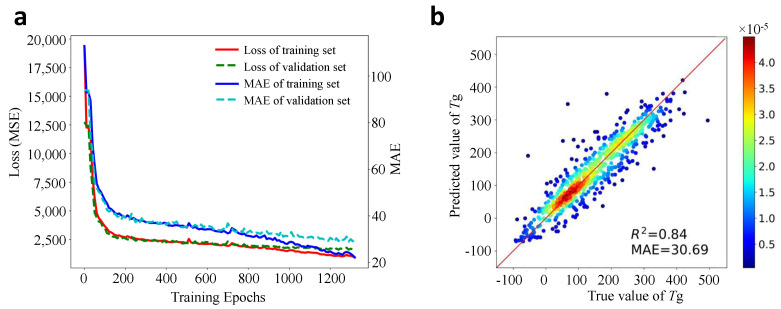
DL model development and evaluation. (**a**): the learning curves of the loss and the MAE with training epochs on the training and validation dataset; (**b**): the performance evaluation of the model on the unseen test dataset (color denotes the density of points).

The learning curve in the training process is shown in Figure 5a. As can be seen from this curve that comparable performances have been achieved on the training and validation dataset. It should be noted that since a patience length of 10 epochs during training is applied, the best model is saved due to early stopping rather than the model trained at the final epoch.

### 3.2. Predictions of the Chemical Language Processing Model on Unseen Data

To further validate the trained chemical language processing model, we apply it to predict $T_{g}$ values of the test dataset. Note that the test dataset is unseen during the training of the DL model. Therefore, the predictability of the DL model can be directly evaluated on this additional dataset, which has 724 polymer samples in total.

After the DL model is well-trained, new predictions can be made easily on the test dataset. The correlation coefficient $R^{2}$ score and the MAE can then be calculated based on the predicted and true values of $T_{g}$, which is plotted in Figure 5b. One can see that the majority of the scatter points locates in the unity red line, indicating the predicted $T_{g}$ values are close to their true values. Quantitatively, the developed DL model gives a correlation score $R^{2}=0.84$ and MAE = 30.69 °C. This performance is reasonably well and comparable with many other ML models for $T_{g}$ prediction in terms of MAE values or $R^{2}$ score [24,37,38,39,79], which confirms the effectiveness of the chemical language processing model. Note that in most previous works, the polymer samples were not large and only certain types of polymers were studied [36], the MAE and $R^{2}$ score may be higher. While in this work, the data size is very large and the types of polymers in the database are very general.

### 3.3. Application of the Chemical Language Processing Model for High-Throughput Screening

**Figure 6:**
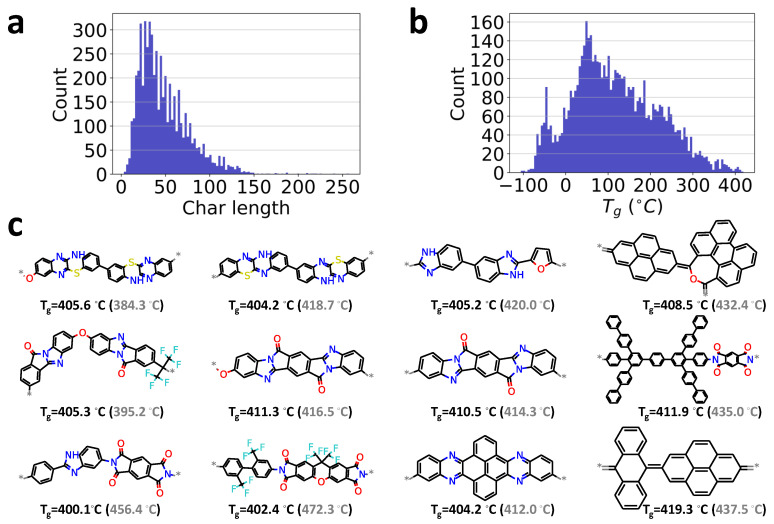
Data visualization for high-throughput screening of high-temperature polymers. (**a**): the distribution of the length of chars in the SMILES strings of polymer’s repeat units; (**b**): the distribution of predicted $T_{g}$ values in the database; (**c**): the 12 candidates with $T_{g}$ larger than 400 °C in the screening of the unlabeled database. ‘*’ indicates the polymerization point in the repeat unit of polymer. Values in the parentheses by gray color are respective $T_{g}$ obtained by MD simulations.

To demonstrate the capability of our chemical language processing model, another unlabeled polymer dataset of 5686 samples without reported $T_{g}$ values are considered for a high-throughput screening task. This dataset collected from earlier work [36] is also from the PolyInfo database [48]. Thus, these two databases are considered similar. It can also be seen from the char length distribution shown in Figure 6a, as compared to the labeled database given in Figure 2b.

To make $T_{g}$ predictions, the polymer’s repeat units in the unlabeled database are first converted into the integer-encoded vector form and then feed into the developed chemical language processing model. The glass transition temperature $T_{g}$ for those unlabeled polymers can be quickly predicted. Figure 6b presents the distribution of the predicted glass transition temperatures $T_{g}$.

For high-throughput screening tasks, the candidates with extreme properties are usually desired and of great value in material discovery [80,81]. As an example, twelve candidates in this unlabeled database with $T_{g}$ larger than 400 °C are quickly identified, as shown in Figure 6c, although their $T_{g}$ values have not been reported before. Particularly, we find the chemical structures of these identified polymers share similar features as other high-temperature polymers, such as polyaryletherketone and polyimide. For instance, saturated 4,5 member rings, bridged rings, benzene rings, oxolane groups, amine groups, and halogens had a higher occurrence rate for polymers with high $T_{g}$ [81,82,83]. For preliminary validation of ML predictions, we have performed all-atom molecular dynamics (MD) simulations on these polymers, with simulation protocols and detailed results given in the Supporting Information. Overall, the $T_{g}$ values predicted from molecular dynamics simulations are in good agreement with ML predictions within the range of uncertainty. It indicates that the proposed model can be employed for high-throughput screening tasks if trained well. Besides, the model’s prediction ability is evaluated on anther dataset of 32 conjugated polymers with experimentally reported *T_g_* values [84]. A reasonable prediction is demonstrated and can be found in the Supporting Informations. However, note that these examples are mainly adopted for demonstration purposes of the chemical language processing model. If the unlabeled database is significantly different from the training database with reported $T_{g}$ values, the DL model would do an extrapolation rather than interpolation, which would lead to inaccurate predicted $T_{g}$.

## 4. Discussion

Here, we formulate the forward prediction of polymer’s $T_{g}$ as a chemical language processing problem, leveraging a large polymer database PolyInfo. The utilization of SMILES strings for polymer’s repeat unit as feature representation is made to develop DL models. To encode the SMILES strings for DL model development, a lexicon composed of individual characters following alphabetic order is applied. Since feature representation is of great importance for ML models [30], alternative forms of polymer lexicon can be developed to build superior chemical language processing models. For example, an element lexicon can be developed based on the atomic element, e.g., using ‘Si’ as a lexicon element for silicon instead of ‘S’ and ‘i’.

Additionally, one potential way to improve model performance is to incorporate more chemical domain knowledge into the model. For instance, adding in molecular weight, topological information of polymers, and processing conditions as additional inputs so that the model can reasonably predict $T_{g}$ with better accuracy. This can be realized by, for example, taking advantage of the hidden vector of the RNN. The additional information can be used to initialize the hidden vector. Alternatively, the information can be added by concatenating to the outputs of RNNs. Moreover, focusing on certain types of polymers, e.g., polyolefin, or polyesters, may also potentially improve the model performances. For example, Pugar et. al. considered polyurethane elastomers and applied ML to extract important physicochemical properties governing $T_{g}$ [85]. Leveraging these descriptors, such as electrostatic, hydrogen bonding, and microstructures of the hard segments, in the model can improve ML model performances. Furthermore, the sampling method of the training dataset can also impact the model performances, especially for studies with a small database [86].

There are several advantages of the feature representation adopted in this work. The use of polymer repeat units is more reasonable than that of monomers as the former is a building block of the corresponding polymers, though the use of polymer monomers has been widely adopted in polymer informatics [39,87,88]. This is probably due to the requirements of cheminformatics packages on the SMILES strings that can be processed. Polymer’s monomers can be easily processed to generate molecular descriptors or fingerprints to be used as inputs for ML model development, while polymer’s repeat units with polymerization point ‘[*]’ may not be feasibly processed in many packages. Besides, there is no additional pre-processing needed before ML model development due to the pure SMILES string used as inputs, in contrast to the use of molecular descriptors or fingerprints. Thus, the formulation of polymer’s $T_{g}$ prediction as a chemical language processing might be more beneficial and efficient. This representation will also benefit the development of generative ML models for the inverse molecular design of polymers.

**Figure 7:**
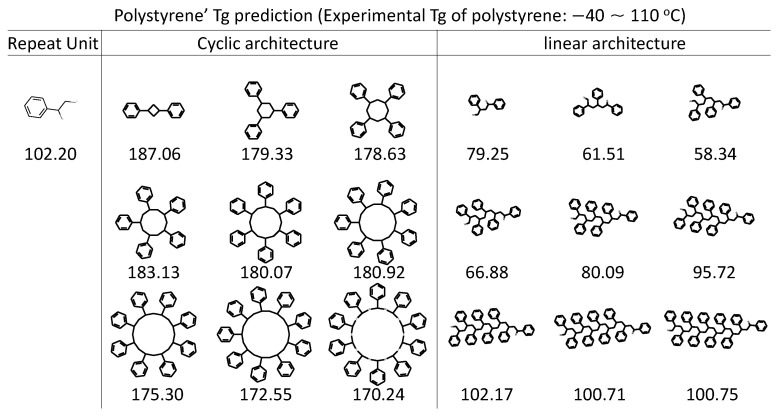
RNN model predictions on various polystyrene architectures. The cyclic architecture and linear architecture of polystyrene being evaluated by the obtained RNN model are accompanied by the $T_{g}$ prediction (in Celsius). Experimental $T_{g}$ is taken from [89], with $T_{g}$ values ranging from −40∼100 °C and 65∼110 °C form linear and cyclic polystyrene polymers, respectively, depending on the molecular weight.

While the polymers in this study are homopolymers, the framework is general and can be extended to study polymer blends of different typologies. The first step is to prepare the inputs which include the SMILES string of composing polymers and the ratio of them. A model is feasible to build from the perspective of ML model development, but the performance remains to be seen depending on the specific system of interest. For example, when polystyrene under cyclic topological constraint is compared with its linear compartment, a reduced hydrodynamic volume has been reported, leading to higher $T_{g}$. Although our RNN model is purely trained on linear polymers, its prediction ability on cyclic architecture is also well demonstrated, as shown in Figure 7. The prediction trend matches well with experiments observation that The cyclic architecture has higher $T_{g}$ compared with the linear analogue [89]. A positive correlation of RNN $T_{g}$ prediction to the molecular weight is well recognized too, especially on the linear architecture which is used for our model training.

## 5. Conclusions

In summary, we proposed a chemical language processing model for predictions of polymer’s $T_{g}$. The SMILES notation of polymer’s repeat unit is adopted as feature representation, which is purely linguistic-based. There are no additional computations needed for pre-processing, in contrast to other conventional polymer informatics models. The key feature of our model is the usage of char embedding and RNN to process the char-based inputs of polymers. Reasonable predictions on polymer’s $T_{g}$ can be achieved using this model. Besides, a high-throughput screening task has been performed on an unlabeled polymer database to identify promising candidates with high $T_{g}$ values that can be used in extreme environments. It suggests that the chemical language processing model may be used as an effective approach to developing predictive ML models for other properties of polymers, such as melting temperature, electronic bandgap, dielectric constant, refractive index, and many others.

## Data Availability

All the polymer data can be found in the PolyInfo database. MD data and code associated with this work are available at the GitHub repository: github.com/figotj/RNN-Tg (accessed on 11 February 2021).

## Supplementary Materials

The following are available online at https://www.mdpi.com/article/10.3390/polym13111898/s1.

## Abbreviations

The following abbreviations are used in this manuscript:

CNN	Convolutional neural network
DL	Deep learning
DNN	Deep neural network
LSTM	Long short-term memory
MD	Molecular dynamics
ML	Machine learning
QSPR	Quantitative structure-property relationships
RNN	Recurrent neural network
SMILES	Simplified molecular-input line-entry system
