# Supplementary material for: Predicting Polymers’ Glass Transition Temperature by a Chemical Language Processing Model
Source: Polymers (Basel). 2021 Jun 7;13(11):1898. doi: 10.3390/polym13111898 (PMC8201381; doi:10.3390/polym13111898)
Supplement: Supplementary file 1 [file polymers-13-01898-s001.zip › polymers-1138769-supplementary.pdf]

Supplementary Material of

Predicting Polymer's Glass Transition Temperature by A Chemical Language Processing Model

Guang Chen[a], Lei Tao[a], Ying Li[a,b]

[a]Department of Mechanical Engineering, University of Connecticut

Storrs, Connecticut, 06269, United States

[b]Polymer Program, Institute of Materials Science, University of Connecticut

Storrs, Connecticut, 06269, United States

Contents

S1: The chemical language processing model development 2

S2: Hyperparameter tuning for SMILES string length of 100 using unidirectional LSTM layer 3

S3: Hyperparameter tuning for SMILES string length of 120 using unidirectional LSTM layer 4

S4: Hyperparameter tuning for SMILES string length of 100 using bidirectional LSTM layer 5

S5: Hyperparameter tuning for SMILES string length of 120 using bidirectional LSTM layer 6

S6: Results with 8/2 data split 7

S7: Tg from MD simulations 8

S8: ML predicted Tg v.s. MD simulated Tg for selected 12 polymers 9

References 12

S1: The chemical language processing model development

The model is developed under the Tensorflow platform [1] and mainly the Keras package [2]. A code example using one bidirectional LSTM layer and a TimeDistributed Dense layer, and a final output layer is shown in Box S1. In all cases, the number of neurons in the dense layer is set as one half of the LSTM hidden units.

Box S1: a code example for the chemical language processing model development.

```
input_dim = 45     # the number of unique tokens
input_len = 100    # the max length of the array (size of inputs)
out_dim = 15       # the output length of the array of the embedding layer
LSTMunits = 40  # the number of hidden neurons used for a LSTM unit

RNNmodel = Sequential()
RNNmodel.add(Embedding(input_dim, out_dim, input_length=input_len))
RNNmodel.add(Bidirectional(LSTM(LSTMunits, return_sequences=True)))
RNNmodel.add(TimeDistributed(Dense(int(LSTMunits/2), activation="relu")))
RNNmodel.add(Reshape((int(LSTMunits/2*input_len),)))
RNNmodel.add(Dense(1))  # the final output layer which predicts Tg
```

Embedding layer

The embedding layer is the first layer of the model. The objective of using an embedding layer is to convert the index-encoded input to a "meaningful" and dense representation during training. As shown in Box S1, it has three parameters. The 'input_dim' is the unique tokens of the chars in the SMILES strings, or the length of the lexicon, which is fixed. The second parameter 'out_dim' is the size of the converted dense vector, which is a hyperparameter that can be tuned. The third parameter 'input_length' is the length of the input, which is also a hyperparameter that can be tuned.

Mathematically, an embedding layer is a transformation matrix, which transforms an input vector into another vector. It not only reduces the dimension of the features, but also learns something meaningful during model training.

S2: Hyperparameter tuning for SMILES string length of 100 using unidirectional LSTM layer

The hyperparameters for model development are the number of LSTM layers, the number of hidden neurons for each LSTM layer, the output dimension of the embedding layer, and the type of intermediate dense layer. The hyperparameter tuning results are shown in Table S1-S4.

Table S1. the evaluation metric (MAE) of the DL models on the unseen test dataset with One LSTM layer with a subsequent dense layer.

		LSTM units					
		10	***20***	***30***	***40***	***50***	***60***
Out dim of embedding layer	***5***	38.04	37.31	35.67	40.38	91.44	36.27
	10	37.18	37.89	38.19	37.11	37.4	35.17
	15	37.27	36.35	38.18	35.64	37.23	35.59
	20	36.98	37.41	37.29	34.44	36.24	32.2
	25	37.65	38.85	38.07	37.45	37.81	33.78

Table S2. the evaluation metric (MAE) of the DL models on the unseen test dataset with One LSTM layer with a subsequent Time Distributed dense layer.

		LSTM units					
		10	***20***	***30***	***40***	***50***	***60***
Out dim of embedding layer	***5***	37.62	36.57	38.36	36.55	36.75	35.56
	10	40.07	36.98	38.11	35.87	37.24	39.37
	15	34.94	35.39	35.76	33.86	36.26	35.22
	20	37.21	33.76	35.93	34.78	34.67	36.19
	25	36.06	34.46	33.69	36.17	32.94	37.88

Table S3. the evaluation metric (MAE) of the DL models on the unseen test dataset with Two LSTM layer with a subsequent dense layer.

		LSTM units					
		10	***20***	***30***	***40***	***50***	***60***
Out dim of embedding layer	***5***	91.42	91.42	91.43	91.46	91.43	91.44
	10	91.44	91.45	91.44	91.43	91.44	91.44
	15	91.42	91.43	91.44	91.45	91.44	91.47
	20	91.42	91.43	91.42	91.44	91.44	91.43
	25	51.44	91.43	91.43	91.47	91.45	91.44

Table S4. the evaluation metric (MAE) of the DL models on the unseen test dataset with Two LSTM layer with a subsequent Time Distributed dense layer.

		LSTM units					
		10	***20***	***30***	***40***	***50***	***60***
Out dim of embedding layer	***5***	38.24	36.78	36.2	35.58	34.05	34.31
	10	36.91	35.74	47.31	33.66	37.29	35.58
	15	36.96	35.07	34.61	36.89	35.9	34.02
	20	37.27	35.03	35.52	35.42	32.76	34.86
	25	35.52	33	34.91	36.9	34.39	34.47

S3: Hyperparameter tuning for SMILES string length of 120 using unidirectional LSTM layer

The hyperparameters for model development are the number of LSTM layers, the number of hidden neurons for each LSTM layer, the output dimension of the embedding layer, and the type of intermediate dense layer. The hyperparameter tuning results are shown in Table S5-S8.

Table S5. the evaluation metric (MAE) of the DL models on the unseen test dataset with One LSTM layer with a subsequent dense layer.

		LSTM units					
		10	20	30	40	50	60
Out dim of embedding layer	5	38.29	91.44	91.44	91.43	91.43	38.57
	10	91.43	39.05	37.92	91.43	37.39	91.45
	15	91.42	37.16	91.43	91.44	91.44	91.46
	20	36.98	38.83	91.43	91.44	35.96	33.81
	25	91.42	91.43	36.81	33.51	36.3	34.32

Table S6. the evaluation metric (MAE) of the DL models on the unseen test dataset with One LSTM layer with a subsequent Time Distributed dense layer.

		LSTM units					
		10	20	30	40	50	60
Out dim of embedding layer	5	37.59	38.42	38.58	35.23	38.18	33.73
	10	35.5	36.39	34.65	42.66	34.84	37.66
	15	35.32	36.75	35.37	34.42	37.12	36.15
	20	39	35.97	35.96	34.15	34.91	34.37
	25	35.46	35.64	35.85	34.29	39.54	34.11

Table S7. the evaluation metric (MAE) of the DL models on the unseen test dataset with Two LSTM layer with a subsequent dense layer.

		LSTM units					
		10	20	30	40	50	60
Out dim of embedding layer	5	91.42	91.43	91.43	91.44	91.44	91.44
	10	91.43	91.42	91.44	91.44	91.45	91.45
	15	91.43	91.43	91.44	91.45	91.44	91.45
	20	91.43	91.43	91.43	91.45	91.43	91.43
	25	91.42	91.43	91.44	91.44	91.43	91.45

Table S8. the evaluation metric (MAE) of the DL models on the unseen test dataset with Two LSTM layer with a subsequent Time Distributed dense layer.

		LSTM units					
		10	20	30	40	50	60
Out dim of embedding layer	5	35.72	37.88	38.35	34.84	37.48	38.36
	10	36.44	35.74	34.57	35.49	38.77	31.47
	15	35.77	37.23	33.5	32.49	36.82	34.83
	20	38.33	35.17	34.2	34.2	37.54	31.09
	25	37.44	33.75	33.46	31.94	34.17	35.3

S4: Hyperparameter tuning for SMILES string length of 100 using bidirectional LSTM layer

The hyperparameters for model development are the number of LSTM layers, the number of hidden neurons for each LSTM layer, the output dimension of the embedding layer, and the type of intermediate dense layer. The hyperparameter tuning results are shown in Table S9-S12.

Table S9. the evaluation metric (MAE) of the DL models on the unseen test dataset with One LSTM layer with a subsequent dense layer.

		LSTM units					
		10	20	30	40	50	60
Out dim of embedding layer	5	36.34	39.07	91.44	82.06	84.06	35.03
	10	36.41	36.75	37.12	35.20	35.67	35.63
	15	36.24	36.02	36.45	35.66	36.15	91.48
	20	36.23	34.83	36.92	36.50	35.55	38.34
	25	35.90	36.00	34.59	35.31	35.66	34.00

Table S10. the evaluation metric (MAE) of the DL models on the unseen test dataset with One LSTM layer with a subsequent Time Distributed dense layer.

		LSTM units					
		10	20	30	40	50	60
Out dim of embedding layer	5	38.22	37.14	38.14	36.03	36.96	36.00
	10	37.79	34.47	37.33	34.88	35.32	38.48
	15	38.30	37.94	34.55	34.79	33.21	34.04
	20	36.13	37.65	36.27	38.77	35.05	34.38
	25	33.89	38.22	34.88	34.47	32.87	35.91

Table S11. the evaluation metric (MAE) of the DL models on the unseen test dataset with Two LSTM layer with a subsequent dense layer.

		LSTM units					
		10	20	30	40	50	60
Out dim of embedding layer	5	39.50	91.43	91.46	91.45	91.47	91.48
	10	36.69	91.44	38.47	91.49	91.44	91.46
	15	36.33	91.43	34.41	91.44	91.47	91.48
	20	36.16	36.47	91.44	91.43	36.24	91.44
	25	36.09	91.42	33.89	32.43	91.45	91.50

Table S12. the evaluation metric (MAE) of the DL models on the unseen test dataset with Two LSTM layer with a subsequent Time Distributed dense layer.

		LSTM units					
		10	20	30	40	50	60
Out dim of embedding layer	5	39.65	35.21	37.35	35.63	36.38	35.59
	10	36.83	35.22	39.58	35.61	36.66	34.06
	15	35.42	37.40	34.11	37.37	35.49	35.81
	20	35.60	33.51	34.16	35.86	35.89	32.93
	25	37.10	34.60	32.66	34.23	34.25	34.74

S5: Hyperparameter tuning for SMILES string length of 120 using bidirectional LSTM layer

The hyperparameters for model development are the number of LSTM layers, the number of hidden neurons for each LSTM layer, the output dimension of the embedding layer, and the type of intermediate dense layer. The hyperparameter tuning results are shown in Table S13-S16.

Table S13. the evaluation metric (MAE) of the DL models on the unseen test dataset with One LSTM layer with a subsequent dense layer.

		LSTM units					
		10	***20***	***30***	***40***	***50***	***60***
Out dim of embedding layer	***5***	37.57	35.56	91.46	91.44	91.46	91.47
	10	37	34.6	91.44	36.49	91.44	36.86
	15	36.86	34.28	36.61	34.31	91.43	37.09
	20	35.99	36.06	35.94	91.45	31.42	91.48
	25	34.95	91.43	35.24	36.62	35.41	36.45

Table S14. the evaluation metric (MAE) of the DL models on the unseen test dataset with One LSTM layer with a subsequent Time Distributed dense layer.

		LSTM units					
		10	***20***	***30***	***40***	***50***	***60***
Out dim of embedding layer	***5***	38.35	37.61	35.27	37.43	38.56	38.51
	10	37.37	35.04	37.3	34.97	37	35.64
	15	36.79	36.72	34.83	35.28	37.02	35.55
	20	35.78	35.78	33.97	38.55	32.45	31.73
	25	37.16	33.82	36.34	37.01	35.67	34.43

Table S15. the evaluation metric (MAE) of the DL models on the unseen test dataset with Two LSTM layer with a subsequent dense layer.

		LSTM units					
		10	***20***	***30***	***40***	***50***	***60***
Out dim of embedding layer	***5***	91.43	91.44	91.45	91.44	91.5	91.49
	10	37.07	91.43	91.44	91.44	91.44	91.46
	15	34.87	91.45	91.45	91.44	91.46	91.47
	20	35.99	91.42	91.44	91.44	91.45	91.49
	25	33.65	33.99	91.44	91.44	91.46	91.5

Table S16. the evaluation metric (MAE) of the DL models on the unseen test dataset with Two LSTM layer with a subsequent Time Distributed dense layer.

		LSTM units					
		10	***20***	***30***	***40***	***50***	***60***
Out dim of embedding layer	***5***	40.38	38.55	32.35	38.26	36.52	35.8
	10	36.48	35.56	35.07	35.62	32.48	34.42
	15	35.28	33.54	36.51	35.07	35.51	30.69
	20	36.48	32.98	31.75	34.71	36.09	38.59
	25	35.19	35	34.97	34.73	33.53	31.44

S6: Results with 8/2 data split

In the main text, we applied 9/1 ratio split because we have a relatively large amount of data, and it is an empirical rule to use 9/1 compared to 8/2. The reason is that one has to ensure the test dataset has enough data points for additional test. For example, if one has 1,000 data points in total, uses of 8/2 is better (200 test data points); while if he/she has 5,000 data points, used of 9/1 is OK (500 test data points). But for completeness, we also add the results using 8/2 split ratio. The model is robust in these two different split cases as well as the other cases reported in the supporting information, as shown below. One can see that comparable model performance is achieved.

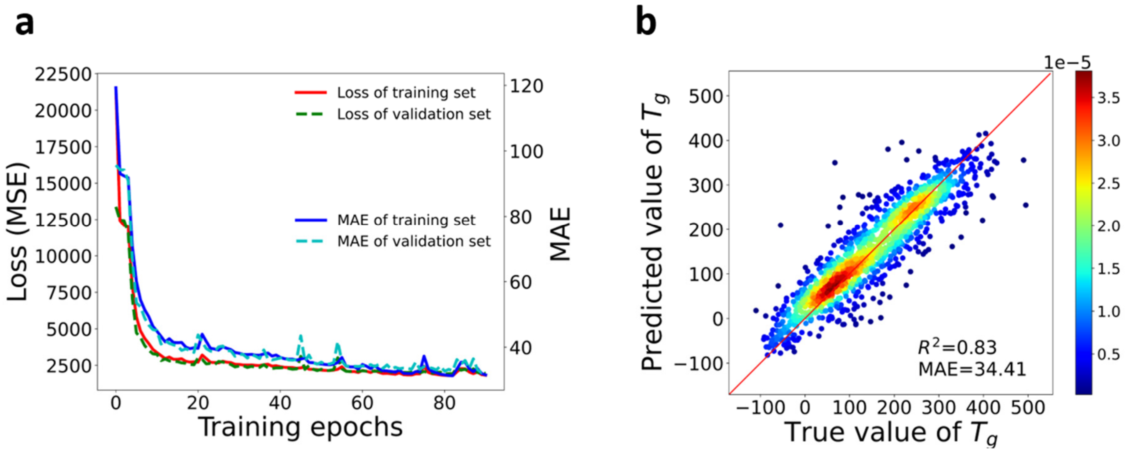

Figure S1. results using 8/2 data split ratio.

S7: Tg from MD simulations

The polymer consistent force field (PCFF) is used to define interatomic interactions for these molecular models of polymers. PCFF is a second-generation force field, which has been parameterized against a wide range of experimental observables for organic compounds containing H, C, N, O, S, P, halogen atoms and ions [3-5]. PCFF has a broad coverage of organic polymers, in calculations of cohesive energies, mechanical properties, compressibilities, heat capacities, elastic constants. For linear polymers, a polymer chain is first built with 20 repeating units connected head-to-tail. After minimizing its energy, 60 polymer chains are used to construct a 3D-periodic amorphous cell, as a representative volume element (RVE). The configuration of the molecules is adjusted in a Monte Carlo fashion. Self-avoiding random walks in space are used to minimize close contacts between atoms, while ensuring a realistic distribution of torsion angles. A homogeneously packed cell is constructed as the linear polymer model. Using LAMMPS (Large-scale Atomic/Molecular Massively Parallel Simulator) package, polymer models are equilibrated first with a 21-step molecular dynamics equilibration protocol [6].

To quantify the glass transition temperature T_g, we apply MD cooling process simulations to each selected polymer models under the isobaric-isothermal ensemble (NPT) from 1000 K to 100 K for 20 nanoseconds. The time step is 1 femtosecond, and the pressure is 1 atmosphere. The obtained specific volume vs. temperature curves are shown in Figure S1. Based on the curve, rubbery and glassy phases are represented by segments of the constant slope. The intersection of the two lines from the least-square line fit indicates the T_g. The cooling rate in MD simulations is much faster than in the experiments considering the nanosecond time scale in MD. Although the faster cooling rate results in a higher T_g, the MD simulated T_g is still proven to be close to the experimental values.

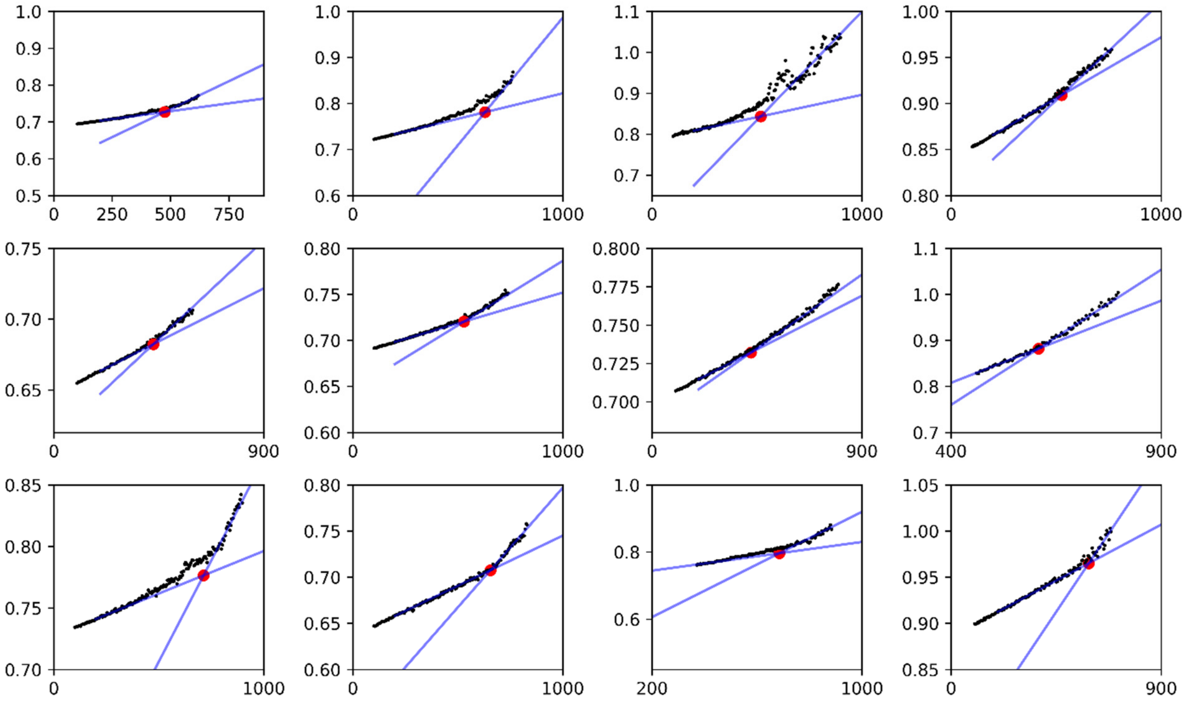

Figure S2. Plots of specific volume v.s. temperature for the 12 selected polymers.

In addition to the selected 12 high Tg polymers, 554 more homopolymers (selected through K-means classification from unlabeled polymer dataset of 5,686 samples without reported Tg values) are simulated using similar MD simulation strategy such that a good amount of data points is available for analysis. Therefore, there are 566 MD simulations in total based on which the RNN model can be better validated on the unlabeled polymer dataset.

S8: ML predicted T_g v.s. MD simulated T_g for validation polymers

The direct comparison of the RNN model prediction and the MD simulation for the selected 12 high Tg polymers are listed in Table S17. Further validating using the whole 566 MD simulation is shown in Figure S3.

Table S17. T_g of the selected polymers from MD simulation and ML prediction. (in Celsius)

	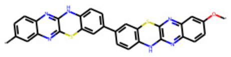	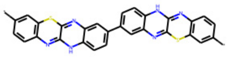	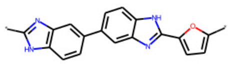	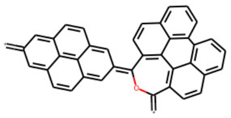
MD	383.40	418.69	419.98	432.43
ML	405.60	404.23	405.18	408.52
	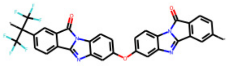	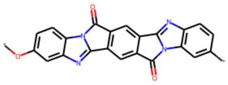	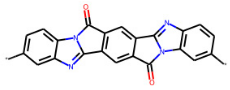	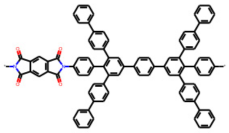
MD	395.15	416.53	414.33	435
ML	405.28	411.27	410.50	411.93
	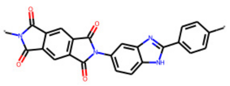	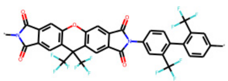	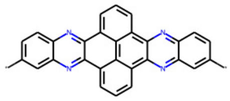	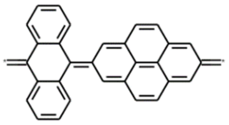
MD	456.35	472.25	411.97	437.49
ML	400.06	402.45	404.18	419.26

We can see that when the RNN model is examined against the MD simulation of 566 homopolymers, the result shows the R^2 = 0.59, the mean absolute error (MAE) = 54.02, and the root-mean-square error (RMSE) = 71.03. It demonstrates a good generalization ability of the RNN model on the unlabeled dataset.

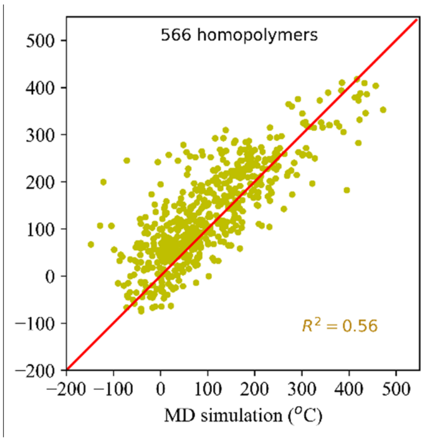

Figure S3. RNN model prediction vs. MD simulation on 566 homopolymers, selected through K-means classification from unlabeled polymer dataset of 5,686 samples without reported Tg values.

The direct comparison of the RNN model prediction and the experimental values for the 32 conjugated polymers is listed in Table S18, taken from Ref. [7]. The mean absolute error (MAE) = 58.29, and the root-mean-square error (RMSE) = 69.94 also demonstrate a reasonable prediction performance.

Table S18. T_g of the 32 conjugated polymers from experiment and ML prediction. (in Celsius)

Polymer	Experiment Tg	Prediction Tg	Polymer	Experiment Tg	Prediction Tg
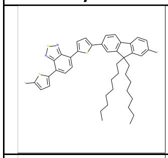	126	195	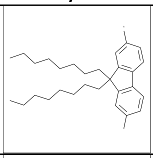	71	52
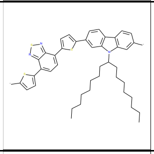	116	201	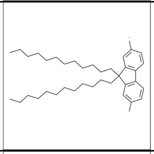	16	38
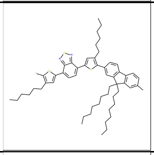	79	214	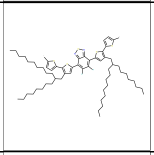	28	134
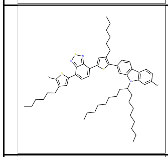	74	214	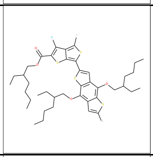	40	167
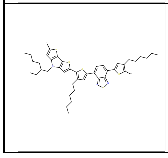	90	215	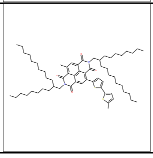	74	85

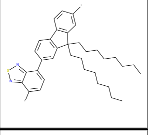	112	180	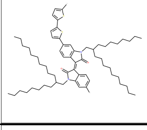	65	101
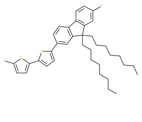	102	195	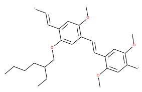	113	148
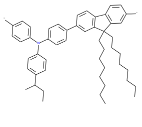	140	175	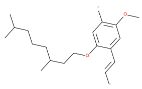	50	81
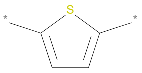	215	102	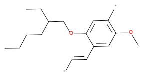	66	98
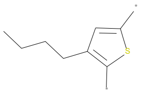	50	82	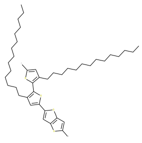	5	64
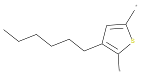	10	70	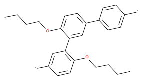	164	219
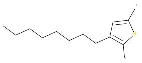	-17	48	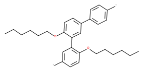	117	159
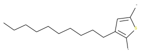	-27	45	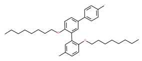	87	113
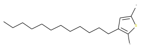	-30	44	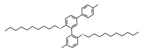	58	74
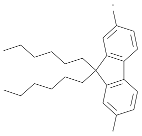	101	62	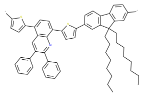	142	160
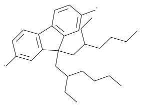	59	59	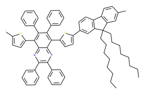	192	166

References

[1]. M. Abadi, P. Barham, J. Chen, Z. Chen, A. Davis, J. Dean, M. Devin, S. Ghemawat, G. Irving, M. Isard, et al., Tensorow: A system for large scale machine learning, in: 12th {USENIX} symposium on operating systems design and implementation ({OSDI} 16), 2016, pp. 265-283.

[2]. F. Chollet, et al., Keras, 2015. URL: https://keras.io/

[3]. Sun, Huai, Pengyu Ren, and J. R. Fried. "The COMPASS force field: parameterization and validation for phosphazenes." Computational and Theoretical Polymer Science 8, no. 1-2 (1998): 229-246.

[4]. Sun, Huai. "COMPASS: an ab initio force-field optimized for condensed-phase applications overview with details on alkane and benzene compounds." The Journal of Physical Chemistry B 102, no. 38 (1998): 7338-7364.

[5]. Sun, Huai, Zhao Jin, Chunwei Yang, Reinier LC Akkermans, Struan H. Robertson, Neil A. Spenley, Simon Miller, and Stephen M. Todd. "COMPASS II: extended coverage for polymer and drug-like molecule databases." Journal of molecular modeling 22, no. 2 (2016): 47.

[6]. Abbott, Lauren J., Kyle E. Hart, and Coray M. Colina. "Polymatic: a generalized simulated polymerization algorithm for amorphous polymers." Theoretical Chemistry Accounts 132, no. 3 (2013): 1334.

[7]. Xie, Renxuan, Albree R. Weisen, Youngmin Lee, Melissa A. Aplan, Abigail M. Fenton, Ashley E. Masucci, Fabian Kempe et al. "Glass transition temperature from the chemical structure of conjugated polymers." Nature Communications 11, No. 1 (2020): 893.
